# Outcomes After a Single Ovarian Stimulation Cycle in Women of Advanced Reproductive Age: A Retrospective Analysis

**DOI:** 10.3389/fendo.2022.792159

**Published:** 2022-02-14

**Authors:** Mengdi Liu, Xusheng Zhao, Yuanyuan Peng, Jiahua Zheng, Kaixuan Guo, Yanli Fan, Lei Jiang, Aimin Yang, Na Cui, Guimin Hao, Wei Wang

**Affiliations:** ^1^ The Second Hospital of Hebei Medical University, Shijiazhuang, China; ^2^ Cangzhou People’s Hospital, Cangzhou, China; ^3^ Handan Women and Children’s Hospital, Handan, China

**Keywords:** advanced reproductive age, assisted reproductive technology, *in vitro* fertilization, intracytoplasmic sperm injection, live birth

## Abstract

**Objectives:**

Previous studies showed that age is the most important factor that determines the outcome after embryo transfer (ET), with either *in vitro* fertilization (IVF) or intracytoplasmic sperm injection (ICSI), regardless whether fresh or frozen cycles. The average cumulative live birth rate (CLBR) following a single ovarian stimulation cycle in women of advanced reproductive age (≥38 years of age) has been reported to be 22.6–34.1%. The purpose of this study is to compare the CLBR after a single ovarian stimulation cycle in women of different advanced reproductive age bracket (38/39, 40/41, 42/43 years of age or older), and to explore the factors (e.g., age, type of infertility, body mass index (BMI), ovarian stimulation protocols) associated with CLBR.

**Methods:**

This retrospective analysis included all women of advanced reproductive age (38 years or older) undergoing IVF or ICSI at authors’ institute during a period from January 1, 2016 to December 31, 2018. The study protocol was approved by the Ethics Committee of the Second Hospital of Hebei Medical University (No. 2021-P045). Subjects with underlying diseases were excluded from analysis. The last follow-up was conducted in December 2020, with minimal 2-year follow-up.

**Results:**

The final analysis included 826 women (40.00 ± 2.10 years of age at the time of ovarian stimulation; n = 633 and 193 for IVF-ET and ICSI-ET, respectively). The number of women in each age bracket was: 424 for 38/39 y, 226 for 40/41 y, 118 for 42/43 y, and 58 for ≥44 y. The number of transferable embryos was 2 (interquartile range: 2,4) for 38/39 y, 2 (2,3) for 40/41 y, 2 (2,3) for 42/43 y, and 2 (1.75,3) for ≥44 y. The rate of fresh embryo transfer was comparable (62.03–72.58%) among the 4 age brackets. The average CLBR following a single cycle was 26.27% in the overall study population, 32.31% for 38/39 y, 26.99% for 40/41 y, 14.4% for 42/43 y, and 3.44% for ≥44 y (*P <*0.001). In multivariate regression, CLBR was independently associated with younger age (OR for each year: 1.538, 95%CI: 1.193, 1.984) and higher number of transferable embryos (OR for each embryo: 1.495, 95%CI: 1.297, 1.722). CLBR differed significantly in the 38/39 group (*P* = 0.014), with higher rate in women receiving the Gonadotropin-releasing hormone agonist (GnRH-a) long or GnRH-a ultra-long protocols.

**Conclusions:**

Forty-two years of age seemed to be a critical cutoff to achieve reasonable level of CLBR after a single ovarian stimulation cycle in women of advanced reproductive age.

## Introduction

Age is the most important factor that determines the outcome after embryo transfer (ET), with either *in vitro* fertilization (IVF) or intracytoplasmic sperm injection (ICSI), regardless whether fresh or frozen cycles (1). Previous studies showed that 38 years of age represent a significant turning point in the decline of cumulative live birth rate (CLBR) after IVF/ICSI-ET (2, 3). The average CLBR following a single ovarian stimulation cycle in women of advanced reproductive age (≥38 years of age) has been reported to be 22.6–34.1% (4, 5). Within this age group, more detailed information is needed in order to formulate an evidence-based strategy to guide decision making by both practitioners and patients. Towards this goal, we conducted a retrospective analysis to compare key outcome measures after IVF/ICSI-ET in women of 38 years of age or older. CLBR after a single ovarian stimulation cycle was compared among the 38/39, 40/41, 42/43 and ≥44 year age brackets. A multivariate regression analysis was conducted to explore the factors (e.g., age, type of infertility, body mass index (BMI), and ovarian stimulation protocol) that are associated with CLBR.

## Methods

### Study Subjects

In this retrospective analysis, we screened all women of 38 years of age or older who underwent IVF/ICSI-ET in the Reproductive Medicine Department of the Second Hospital of Hebei Medical University during a period from January 1, 2016 to December 31, 2018. The last follow-up was conducted in December 2020, with a minimal 2-year follow-up.

For inclusion in analysis, subjects must be at least 38 years of age at the time of ovarian stimulation and received at least one ET cycle. Subjects with one or more of the following conditions were excluded from the analysis: 1) chromosomal abnormality; 2) preimplantation genetic testing; 3) transfer of embryos obtained from multiple ovarian stimulation cycles; 4) oocyte cryopreservation; 5) egg donation; 6) underlying endocrine diseases (for example, hyperprolactinemia, hyperthyroidism, polycystic ovary syndrome); 7) intrauterine adhesion; 8) malformation, tuberculosis or history of surgery of the reproductive system; 9) any type of autoimmune diseases; 10) history of recurrent miscarriage or repeated implantation failure; and 11) nature cycles. Subjects with used embryo(s) but no live birth during the study period were also excluded from the analysis.

### Ovarian Stimulation and Embryo Transfer

Ovarian stimulation protocols included gonadotrophin-releasing hormone agonist (GnRH-a) long protocol, GnRH-a ultra-long protocol, GnRH antagonist protocol, progestin primed ovarian stimulation and micro-stimulation protocol. Protocol selection was based on physician discretion. Ovulation was triggered with human chorionic gonadotropin (hCG) when the dominant follicle(s) reached 18 mm in diameter and E2 level at 150–300 ng/L per mature follicle (6–8). The oocytes were retrieved under the guidance of vaginal ultrasound 36–38 h after hCG injection, and fertilized using conventional IVF or ICSI based on semen analysis. For fresh ET, cleavage embryos (3 day) were used. Embryo quality was assessed using the Bourn Hall Clinic criteria for fresh embryos (grade I or II). For frozen ET, hormone replacement cycles were used for endometrial preparation.

### Outcome Measures

Biochemical pregnancy was defined by positive blood β-HCG (≥50 mIU/ml) at 14 day after ET. Clinical pregnancy was defined as at least one gestational sac in the uterus. CLBR is presented as live birth episodes per patient per oocytes retrieval to account for the first live birth (9).

### Statistical Analysis

Continuous variables with normal distribution are presented as mean ± standard deviation (SD), with Bonferroni test for posthoc pairwise comparison among different age brackets. Continuous variables with skewed distribution are presented as median (Q1, Q3), analyzed using the Kruskal–Wallis H test, with Bonferroni test for posthoc pairwise comparison. Categorical variables are presented as number and percentage, and analyzed using the chi-square or Fisher’s exact probability test, as appropriate. A multivariate logistic regression analysis was conducted to examine the factors associated with CLBR. Candidate factors in the multivariate regression were selected based on: 1) factors that known to be associated with live birth (such as age and basal follicle-stimulating hormone (FSH)); 2) univariate analysis that examined the association of each factor with cumulative live birth, at a threshold of *P <*0.1. Statistical significance was defined as *P <*0.05 (2-sided). All analyses were conducted using SPSS24.0 statistical software.

## Results

**Figure 1:**
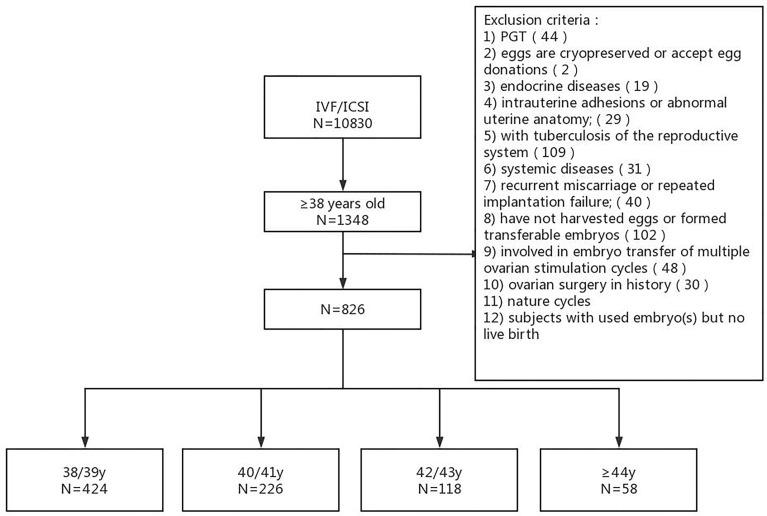
Study flow chart.

The analysis included 826 women (40 ± 2.10 years of age at the time of ovarian stimulation) and 996 ET cycles (553 and 443 for fresh and frozen ET cycles, respectively) (**Figure 1**). The number of women in each age bracket was: 424 for 38/39 y, 226 for 40/41 y, 118 for 42/43 y, and 58 for ≥44 y. The rate of primary infertility in each age bracket was: 24.29% (103/424) for 38/39 y, 20.35% (46/226) for 40/41 y, 18.64% (22/118) for 42/43 y, and 15.51% (9/58) for ≥44 y. The number of IVF-ET and ICSI-ET was 633 and 193, respectively. The number of transferred embryos was either 1 or 2. The number of ET cycles was 1 in 678 women (553 and 125 for fresh and frozen cycles, respectively), 2 in 129 women (a fresh ET cycle plus a frozen ET cycle in 90 women), and 3 in 17 women (a fresh ET cycle followed by 2 frozen ET cycle in 9 women).

### Baseline Characteristics

Antral follicle count (AFC) was 9 (6,12) for 38/39 y, 8 (6,11) for 40/41 y, 6 (4.5,10) for 42/43 y, and 5 (4,7) for ≥44 y (*P <*0.001). Pairwise comparison showed higher AFC in the 38/39 y group than in the 42/43 y and ≥44 y groups (*P <*0.001 for both), and higher AFC in the 40/41 y group than in the 42/43 y and ≥44 y groups (*P* = 0.003 and <0.001, respectively). There was no statistically significant difference between the 38/39 y and 40/41 y groups (*P* = 0.490), and between the 42/43 y and ≥44 y groups (*P* = 0.711). There was a significant trend for increasing basal FSH with increasing age (*P* = 0.008). FSH in the 38/39 y group was significantly higher than in the ≥44 y (*P* = 0.033), but there was no significant difference between any other groups. The four groups did not differ in type of infertility, years of infertility, BMI, endometrium thickness and other baseline hormone levels.

### Ovarian Stimulation Protocols

The percentage of specific ovarian protocol was 37.41% (309/826) for GnRH antagonist protocol, 26.63% (220/826) for GnRH-a long protocol, 21.31% (176/826) for micro-stimulation protocol, 12.35% (102/826) for GnRH-a ultra-long protocol, 2.30% (19/826) for progestin primed ovarian stimulation. Percentage of GnRH antagonist protocol and GnRH-a long protocol and GnRH-a ultra-long protocol differed significantly among the 4 age brackets (*P <*0.001, respectively, **Table 1**).

**Table 1:** Ovarian stimulation protocols in different age brackets and data of *in vitro* fertilization and embryo transfer.

	Total	38/39 y	40/41 y	42/43 y	≥44 y	*P*
	N = 826	N = 424	N = 226	N = 118	N = 58	
GnRH antagonist	37.41% (309/826)	31.83% (135/424)	35.84% (81/226)	47.46% (56/118)	63.79% (37/58)	<0.001
GnRH-a long	26.63% (220/826)	33.49% (142/424)	20.80% (47/226)	22.03% (26/118)	8.62% (5/58)	<0.001
Micro-stimulation	21.31% (176/826)	18.16% (77/424)	25.66% (58/226)	23.73% (28/118)	22.41% (13/58)	1.38
GnRH-a ultra-long	12.35% (102/826)	15.09% (64/424)	14.16% (32/226)	4.24% (5/118)	1.72% (1/58)	<0.001
Progestin primed ovarian stimulation	2.30% (19/826)	1.42% (6/424)	3.54% (8/226)	2.54% (3/118)	3.44% (2/58)	0.335
Number of oocytes	6 (3,10)	7 (4,10)	6 (4,10)	5 (3,8)	3 (2,5.25)	<0.001
Number of transferable embryos						
1	130	12.50% (53/424)	15.93% (36/226)	22.88% (27/118)	24.14% (14/58)	0.012
2	342	46.46% (197/424)	34.51% (78/226)	36.44% (43/118)	41.37% (24/58)	0.018
3	160	13.21% (56/424)	27.43% (62/226)	23.73% (28/118)	24.14% (14/58)	<0.001
4–11	194	27.83% (118/424)	22.12% (50/226)	16.95% (20/118)	10.34% (6/58)	0.004
Number of transplant cycles						
1	678	77.12% (327/424)	86.28% (195/226)	87.29% (103/118)	91.38% (53/58)	0.002
2	129	20.75% (88/424)	10.62% (24/226)	10.17% (12/118)	8.62% (5/58)	0.001
≥3	19	2.12% (9/424)	3.10% (7/226)	2.54% (3/118)	0.0% (0/58)	0.553

### *In Vitro* Fertilization and Embryo Transfer

The number of oocytes collected was 7 (4,10) for 38/39 y, 6 (4,10) for 40/41 y, 5 (3,8) for 42/43 y, and 3 (2,5.25) for ≥44 y (*P <*0.001; **Table 1**). Pairwise comparison showed higher number of oocytes in the 38/39 y and 40/41 y groups than in the 42/43 y and ≥44 y groups. There was no statistically significant difference between the 38/39 y and 40/41 y groups (*P* = 1.0), and between the 42/43 y and ≥44 y groups (*P* = 0.155). The number of transferable embryos was lower with increasing age.

### Cumulative Live Birth

The CLBR was 32.31% (137/424) in the 38/39 y group, 26.99% (61/226) for 40/41 y, 14.40% (17/118) for 42/43 y, and 3.44% (2/58) for ≥44 y (*P <*0.001; **Table 2**). The CLBR in the 38/39 y and 40/41 y groups were significantly higher than in the 42/43 y and ≥44 y groups (*P <*0.001 for 38/39 y vs. 42/43 y and ≥44 y, *P* = 0.008 and *P <*0.001 for 40/41 y vs. 42/43 y and ≥44 y).

**Table 2:** Cumulative live birth after a single ovarian stimulation cycle and pregnancy outcomes in first ET cycles.

	Total	38/39 y	40/41 y	42/43 y	≥44 y	*P*
	N = 826	N = 424	N = 226	N = 118	N = 58	
Cumulative live birth	26.27% (217/826)	32.31% (137/424)	26.99% (61/226)	14.40% (17/118)	3.44% (2/58)	<0.001
Fresh ET cycle	66.95% (553/826)	62.03% (263/424)	67.70% (153/226)	77.97% (92/118)	77.58% (45/58)	0.003
Clinical pregnancy rate (%)	26.15% (216/826)	29.48% (125/424)	29.20% (66/226)	17.80% (21/118)	6.89% (4/58)	<0.001
Live birth rate(%)	21.43% (177/826)	25.94% (110/424)	22.57% (51/226)	11.86% (14/118)	3.44% (2/58)	<0.001
Abortion rate(%)	24.54% (53/216)	17.60% (22/125)	31.82% (21/66)	38.10% (8/21)	50.00% (2/4)	0.035
Multiple pregnancy rate (%)	13.43% (29/216)	16.80% (21/125)	9.09% (6/66)	9.52% (2/21)	25.00% (1/4)	0.407

### Pregnancy Outcomes in First ET Cycles

For the first ET cycles, the percentage of fresh ET was 62.03% (263/424) in 38/39 y, 67.70% (153/226) in 40/41 y, 77.97% (92/118) in 42/43 y, and 77.58% (45/58) in ≥44 y groups (*P* = 0.003, **Table 2**). Clinical pregnancy rate in the 38/39 y group (29.48%) and 40/41 y group (29.20%) was higher than in the ≥44 y groups (6.89%) (*P <*0.001, respectively). There was no difference between the 38/39 y and 40/41 y groups (*P* = 0.941) and between the 42/43 y and ≥44 y groups (*P* = 0.052).

Live birth rate (LBR) was 25.94% (110/424) in 38/39 y, 22.57% (51/226) in 40/41 y, 11.86% (14/118) in 42/43 y, and 3.44% (2/58) in ≥44 y groups (*P <*0.001). The 38/39 y group had higher LBR than the 42/43 y and ≥44 y groups (*P* = 0.001 and <0.001, respectively). The 40/41 y group had higher LBR than the ≥44 y groups (*P* = 0.001). Abortion rate differed among the 4 groups (*P* = 0.035), but there was no difference in pairwise comparison. Multiple pregnancy rate did not differ among the 4 groups (*P* = 0.407).

### Cumulative Live Birth of Different Ovarian Stimulation Protocols

CLBR was 22.0% (68/309) in the GnRH antagonist group, 33.2% (73/220) in the GnRH-a long group, 20.5% (36/176) in the micro-stimulation, 38.2% (39/102) in the GnRH-a ultra-long group, and 10.5% (2/19) in the progestin primed ovarian stimulation (*P <*0.001; **Table 3**). CLBR in the GnRH-a long group and the GnRH-a ultra-long group was higher than in the GnRH antagonist group and in the micro-stimulation.

**Table 3:** Cumulative live birth of different ovarian stimulation protocols in different age brackets.-, not analyzed since the number of the subjects is <15.

	Total	38/39y	40/41y	42/43y	≥44y
		N=424	N=226	N=118	N=58
GnRH antagonist	22.0%	27.4%	29.6%	10.7%	2.7%
	(68/309)	(37/135)	(24/81)	(6/56)	(1/37)
GnRH-a long	33.2%	38.7%	23.4%	23.1%	–
	(73/220)	(55/142)	(11/47)	(6/26)	(1/5)
Micro-stimulation	20.5%	22.1%	24.1%	17.9%	–
	(36/176)	(17/77)	(14/58)	(5/28)	(0/13)
GnRH-a ultra-long	38.2%	42.2%	37.5%	–	–
	(39/102)	(27/64)	(12/32)	(0/5)	(0/1)
Progestin primed ovarian stimulation	10.5%	–	–	–	–
	(2/19)	(1/6)	(1/8)	(0/3)	(0/2)
*P*	<0.001	0.014	0.482	0.307	–

### Factors Associated With Cumulative Live Birth

In multivariate logistic analysis, cumulative live birth was independently associated with: younger age (OR for each year: 1.538, 95%CI: 1.193, 1.984) and higher number of transferable embryos (OR for each embryo: 1.495, 95%CI: 1.297, 1.722), and not with BMI (OR: 0.959, 95%CI: 0.895, 1.028), infertility type (OR: 1.191, 95%CI: 0.730, 1.943), AFC (OR for each antral follicle: 0.973, 95%CI: 0.917, 1.033) and ovarian stimulation protocol (OR: 4.146, 95%CI: 0.468, 36.695).

## Discussion

Consistent with previous studies showing decreased LBR with increasing age (4, 10), we found progressively lower CLBR after a single ovarian stimulation cycle in women of advanced reproductive age. The CLBR was 32.31% in the 38/39 y group, 26.99% in the 40/41 y group, 14.40% in the 42/43 y group, and 3.44% in the ≥44 y group. The CLBR in the 38/39 y and 40/41y groups was significantly higher than in the 42/43 and ≥44 y groups, and there seems to be a clinically relevant reduction at 42 years of age.

Among the 26.27% women with live birth, 81.6% (177/217) achieved live birth after only one transplantation cycle. Key measures in the first ET cycle in the 4 age brackets were generally consistent with cumulative measures after a single ovarian stimulation cycle, including lower LBR, clinical pregnancy rate with increasing age.

In multivariate regression analysis, CLBR was independently associated with younger age (OR for each year: 1.538, 95%CI: 1.193, 1.984) and higher number of transferable embryos (OR for each embryo: 1.495, 95%CI: 1.297, 1.722). The findings are consistent with a previous study by Xu and colleagues (11).

The risk for aneuploidy increases with age. The aneuploidy rate increases from 20% at 35 years to approximately 80% at 42 years of age (12). A study by Hogan et al. (5) also revealed significant impact of donor age on CLBR. Another study of Hogan found (13) that women aged ≥40 years old had significantly higher CLBR using donor oocytes than using autologous oocytes, suggesting that oocyte aging is a major contributing factor to the decline in cumulative pregnancy rate with the ageing process (12). Consistently, we found significant reduction of both clinical pregnancy rate and CLBR at 42 years of age in the current study.

Advanced reproductive age is associated with lower ovarian reserve and hyporesponse of the ovaries. In the current study, we found lower number of oocytes collected and transferable embryos with increasing age, particularly at the cutoff of 42 years of age. Such a finding is consistent with the notion by Ferraretti (14) that embryo quality is the most crucial factor that determines the pregnancy outcomes. Previous studies have shown that the number of oocytes obtained is an independent factor in the cumulative birth rate (15). A study based on single ET also demonstrated that higher number of oocytes is associated with higher LBR after using all frozen embryos (16). In older women (>40 years of age) with selective single ET, however, the observation was somewhat different: oocyte number was not a significant factor affecting the cumulative birth rate (17). Higher number of oocytes obtained is almost always accompanied by higher number of transferable embryos and the number of high-quality embryos (18).

Selection of appropriate ovarian stimulation protocol is a challenge in women of advanced reproductive age. The current study suggested higher CLBR in the GnRH-a long group and the GnRH-a ultra-long group. For 38/39 y group, there also seemed to be a difference in the CLBR between the protocols (*P* = 0.014), but pairwise comparison did not reveal statistically significant difference among 4 age groups.

The four groups did not differ in endometrium thickness (*P* = 0. 832, **Table 4**). Other factors that could influence the uterus compatibility with healthy pregnancy (19, 20) were not examined in the current study.

**Table 4:** Baseline characteristics in different age brackets.

	Total	38/39 y	40/41 y	42/43 y	≥44 y	*P*
	N = 826	N = 424	N = 226	N = 118	N = 58	
BMI (kg/m^2^)	23.44 (21.83, 25.90)	23.34 (21.60, 25.70)	23.90 (22.12, 25.99)	23.42 (21.86, 26.04)	24.61 (22.00, 26.73)	0.061
Primary infertility,	180 (21.79%)	103 (24.29%)	46 (20.35%)	22 (18.64%)	9 (15.51%)	0.277
Years of infertility	4 (2,8)	4 (2,8)	4 (2,8)	2.5 (1,6.75)	2 (1,9)	0.229
AFC	8 (6,11)	9 (6,12)	8 (6,11)	6 (4.5,10)	5 (4,7)	<0.001
FSH (mIU/ml)	7.78 (6.49,10.00)	7.62 (6.37,9.74)	7.76 (6.44,9.67)	8.42 (6.82,10.85)	8.95 (7.22,11.30)	0.008
LH (mIU/ml)	3.93 (2.94,5.40)	3.93 (2.92,5.54)	4.11 (2.95,5.26)	3.82 (2.95,5.36)	3.9 (3.00,8.64)	0.989
E_2_ (ng/L)	39.00 (27.00, 59.00)	39.00 (26.50, 55.00)	42.00 (28.00, 61.00)	37.00 (25.00, 59.00)	44.00 (26.50, 69.00)	0.401
AMH (ng/ml)	1.49 (0.90,2.57)	1.74 (1.09,2.71)	1.64 (1.06,2.57)	1.10 (0.52,2.67)	0.83 (0.48,1.25)	0.231
Endometrium thickness (mm)	10 (9,11)	10 (9,11)	10 (9,12)	10 (8.1,11)	10 (9,11)	0.832

A strength in the current study is the use of CLBR after a single ovarian stimulation cycle, which cover all ET cycles (whether fresh or frozen) after a single ovarian stimulation cycle, oocyte retrieval and IVF/ICSI, and therefore represents a comprehensive and pragmatic outcome measure meaningful for decision-making by the patients (21). This study has several limitations. First, the follow-up time was limited to 2 years. Second, the sample size is relatively small considering the multitude of confounding factors. Third, this is a retrospective analysis. As such, the selection of ovarian stimulation protocols and the use of fresh vs. frozen ET were not controlled.

## Conclusion

In women of advanced reproductive age (≥38 years), CLBR after a single ovarian stimulation cycle declines rapidly with advancing age, with 42 years age seemingly a critical point. In addition to age bracket, the number of transferable embryos is independently associated with cumulative live birth after a single ovarian stimulation cycle.

## Data Availability Statement

The raw data supporting the conclusions of this article will be made available by the authors, without undue reservation.

## Ethics Statement

The studies involving human participants were reviewed and approved by the Research Ethics Committee of the second hospital of Hebei Medical University. Written informed consent for participation was not required for this study in accordance with the national legislation and the institutional requirements.

## Author Contributions

WW, ML, XZ, and YP contributed to the conception and design of the study. ML contributed to the manuscript’s writing. XZ, YP, JZ, and KG contributed to the manuscript’s editing. The remaining authors participated in data collection and patients’ follow-up. All authors listed have made a substantial, direct, and intellectual contribution to the work and approved it for publication.

## Funding

This study was supported by the Health Special Project of Science and Technology Department of Hebei Province (21377760D), the Natural Science Foundation of Hebei Province (H2021206377) and the government-funded clinical medicine outstanding talent project in 2021.

## Conflict of Interest

The authors declare that the research was conducted in the absence of any commercial or financial relationships that could be construed as a potential conflict of interest.

## Publisher’s Note

All claims expressed in this article are solely those of the authors and do not necessarily represent those of their affiliated organizations, or those of the publisher, the editors and the reviewers. Any product that may be evaluated in this article, or claim that may be made by its manufacturer, is not guaranteed or endorsed by the publisher.
